# A Comparison of Mental Health Care Systems in Northern and Southern Europe: A Service Mapping Study

**DOI:** 10.3390/ijerph15061133

**Published:** 2018-05-31

**Authors:** Minna Sadeniemi, Nerea Almeda, Jose A. Salinas-Pérez, Mencía R. Gutiérrez-Colosía, Carlos García-Alonso, Taina Ala-Nikkola, Grigori Joffe, Sami Pirkola, Kristian Wahlbeck, Jordi Cid, Luis Salvador-Carulla

**Affiliations:** 1 Department of Social Services and Health Care, City of Helsinki, Southern Psychiatric Outpatient Clinic, Työpajankatu 14, FI-00099 Helsinki, Finland; 2 University of Helsinki and Helsinki University Hospital, Välskärinkatu 12, FI-00029 Helsinki, Finland; taina.ala-nikkola@hus.fi (T.A.-N.); grigori.joffe@hus.fi (G.J.); 3 Unit for Mental Health, National Institute for Health and Welfare (THL); Mannerheimintie 168, FI-00270 Helsinki, Finland; kristian.wahlbeck@thl.fi; 4 PSICOST Research Association, Department of Psychology, Universidad Loyola Andalucía, C/Energía Solar 1, 41014 Sevilla, Spain; nmalmeda@uloyola.es (N.A.); menciaruiz@uloyola.es (M.R.G.-C.); 5 PSICOST Research Association, Department of Quantitative Methods, Universidad Loyola Andalucía, C/Energía Solar 1, 41014 Sevilla, Spain; jsalinas@uloyola.es (J.A.S.-P.); cgarcia@uloyola.es (C.G.-A.); 6 University of Tampere School of Health Sciences, and Tampere University Hospital, Lääkärinkatu 1, FI-33014 Tampere, Finland; sami.pirkola@staff.uta.fi; 7 Mental Health & Addiction Research Group, Institut d’Investigacions Biomèdiques de Girona (IdibGI)-Institut d’Assistència Sanitària, 17190 Salt Girona, Spain; jordi.cid@ias.cat; 8 VIDEA Lab, Centre for Mental Health Research, Australian National University, 63 Eggleston Rd, Acton ACT 2601, Australia; luis.salvador-carulla@anu.edu.au

**Keywords:** mental health services, mental health care, deinstitutionalization, main type of care, standard comparison, community care

## Abstract

Mental health services (MHS) have gone through vast changes during the last decades, shifting from hospital to community-based care. Developing the optimal balance and use of resources requires standard comparisons of mental health care systems across countries. This study aimed to compare the structure, personnel resource allocation, and the productivity of the MHS in two benchmark health districts in a Nordic welfare state and a southern European, family-centered country. The study is part of the REFINEMENT (Research on Financing Systems’ Effect on the Quality of Mental Health Care) project. The study areas were the Helsinki and Uusimaa region in Finland and the Girona region in Spain. The MHS were mapped by using the DESDE-LTC (Description and Evaluation of Services and Directories for Long Term Care) tool. There were 6.7 times more personnel resources in the MHS in Helsinki and Uusimaa than in Girona. The resource allocation was more residential-service-oriented in Helsinki and Uusimaa. The difference in mental health personnel resources is not explained by the respective differences in the need for MHS among the population. It is important to make a standard comparison of the MHS for supporting policymaking and to ensure equal access to care across European countries.

## 1. Introduction

The European health care model builds on the principle of providing equal access to health care. Countries have developed differing models of service provision to ensure the optimal use of resources to provide health care for the population. Mental disorders contribute considerably to the burden of disease for Europeans [1], but mental health systems vary widely from country to country [2]. These contextual differences in health service provision may have a very significant impact on the analysis of the effectiveness and transferability of new health interventions [3], on clinical practice (e.g., impact of mobility on patient’s care) [4], and on regional health policy (e.g., strategies towards the harmonization and equity of care in Europe) [5].

The common mental health strategy for the World Health Organization (WHO) European region, as formulated in the Mental Health Action Plan for Europe [6], is to ensure good access to mental health care by reforming specialized mental health services (MHS), shifting from large institutions to community-based care supported by psychiatric beds in general hospitals. In high income countries, a modern balanced care model includes both outpatient care, mobile services, hospital beds, and housing services [7]. Services are recommended to be provided at the primary care, general mental health care, and specialized mental health care levels [8]. It is of interest to benchmark the degree of deinstitutionalization, the resources invested in community care, and the output of the reformed service provision system in European countries. Standardized comparison data on the structure and personnel resources of the MHS across European countries, however, has been sparse and mainly limited to policy reports.

This study is a part of the EU (European Union) -funded Research on Financing systems’ Effects on the quality of Mental health care (REFINEMENT) project, which included nine European countries: Austria, England, Estonia, Finland, France, Italy, Norway, Romania, and Spain. The countries were chosen on the basis that they represent different ways of organizing the MHS. The study area within the country had the following inclusion criteria: (1) The population of the area was between 200,000 and 1,500,000 inhabitants, (2) the area covered at least one health district but was not limited to a macro-urban area within a municipality, and (3) reliable sources of information on the local MHS were available.

In a recent study from the REFINEMENT project [2], wide differences in the MHS were found between Spain and Finland, although both catchment areas are considered as benchmarking areas and are in many ways comparable. Both Finland and Spain have tax-funded mental health systems, and the care is provided by salaried professionals in groups instead of having a majority of independent single-handed professionals. Having a similar way of financing the MHS but differing in some significant aspects (a Nordic welfare state versus a southern European, family-centered country), it was of interest to study what state had the process of deinstitutionalization reached and how the services were organized. We set out to compare the implementation of deinstitutionalization policies and the productivity of MHS in northern and southern Europe, analyzing one large benchmarking catchment area in Finland and one in Spain.

In Finland, a national mental health policy was formulated for the period 2010–2015. The plan emphasized the development of community MHS, downsizing, and the closure of separate mental hospitals and strengthening mental health capacity in primary care (Ministry of Social Affairs and Health 2010). An evaluation of the reform (Ministry of Social Affairs and Health 2016) found mostly positive impacts, especially a reduction in the compulsion and inclusion of experts by lived experiences in the care system [9].

The municipalities in Finland are responsible for arranging public health care and social services for their residents, and governmental steering is weak. To fund these services, municipalities have the right to levy taxes and to collect out-of-pocket user fees. All residents are covered by public health care. Each municipality is free to provide health and social services as a municipal activity or to purchase the services from an external provider, e.g., another municipality, a joint municipal authority, or even a private provider [10]. Specialized services are, in general, provided by joint municipal authorities, i.e., hospital districts, but large municipalities may also have chosen to produce specialized health care (such as psychiatric services) as a municipal activity [11].

Municipal health centers in Finland provide primary care, including the prevention, diagnosis, and treatment of common mental disorders, such as depression, anxiety disorders, and alcohol use disorders. Most health centers have a specialized mental health care staff, usually psychologists and psychiatric nurses [12]. Depending on the share of work between the health center and the hospital district, some health centers may have mental health units led by psychiatrists. Some health centers may offer day treatment, in addition to outpatient appointments. Access to elective specialized care is usually provided by reference from primary care only. In psychiatric emergencies, direct access to secondary care is possible. In Finland, the deinstitutionalization process has resulted in a partly uncontrolled proliferation of housing services for people with mental health problems. Housing services are mainly owned by private companies or third-sector providers (either for-profit or non-profit), who sell their services to the municipalities [10,13,14].

In Spain, reform of the mental health system started in 1983, with the establishment of the Ministerial Commission for the ‘Psychiatric Reform’ [15], which outlined significant achievements, such as a new organizational structure of care, integration of psychiatric patients in the general health care system, and the creation of an extensive community network of mental health centers. [16]. The application of the reform has followed an uneven course in Spain as a whole, with marked differences between the different autonomous communities. Since 2007, different versions of the Mental Health Strategy of the National Health System [17,18] emphasize the importance of promoting general health in people with severe mental disorders and define a series of action lines to be incorporated and applied by the autonomous communities.

In Spain, the statutory and universal SNS (National Health System) is funded from taxes and predominantly operates within the public sector. Provision is free of charge at the point of delivery. Governance of the health care was totally devolved to the autonomous communities in 2002, which resulted in 17 regional health ministries, with primary jurisdiction over the organization and delivery of health services within their territory. The mental health patients in Spain typically enter the system through primary care [19]. Thirty percent of primary care patients report a mental health problem [20]. Primary care centers fulfill a gatekeeping function, like in Finland, and transfer more severely ill patients to secondary specialized care provided at reference community mental health centers. A minority of users enter the system through emergency care. Improving the coordination of primary care and community mental health care is one of the priorities of the system, and cooperation programs and earmarked funding for coordination activities are currently being implemented.

When developing accessible, affordable, and effective mental health systems, the exchange of data between countries is an important moving force towards better mental health care [21]. Evidence-informed planning and policies requires the analysis and benchmarking of mental health care systems. It is of interest how the mental health service reforms have been implemented in a Nordic welfare state compared to a Mediterranean family-centered society [15].

We set out to compare the adult mental health service provision in northern Europe, represented by the Helsinki and Uusimaa region in Finland (1.2 million adult inhabitants), with the service provision in southern Europe, represented by the Girona region (0.6 million adult inhabitants) in Catalonia, Spain. The aim of the study was to increase the comparative knowledge base of the structure of two divergent mental health systems in Europe. The specific objectives were to compare:

1. The typology and pattern of service availability, according to the main types of care and placement capacity,
2. the personnel resource allocation in different mental health professional groups, and
3. the productivity of specialized services provided per personnel resource.

## 2. Materials and Methods

In Finland, the study area was Helsinki and Uusimaa, and was comprised of 26 municipalities. The Helsinki and Uusimaa area is the most densely populated area in Finland, with 1.5 million inhabitants (1.2 million adults), including the capital city of Helsinki, with over 600,000 inhabitants. The Hospital District of Helsinki and Uusimaa, owned and governed by the municipalities of the region, provided most of the secondary and tertiary health care services, including MHS. Helsinki and Uusimaa consisted of five geographical subareas. Some municipalities, to the widest extent was the capital city of Helsinki, also provided specialized psychiatric services. There were, altogether, eight public psychiatric hospitals in the study area. Municipalities provided primary care MHS at health centers. Non-hospital residential psychiatric services were provided by private companies and the third sector and purchased by the social sector of the municipalities.

The Spanish study area was the Girona Health Region in Catalonia. Catalonia is considered a benchmarking area for integrated care for chronic illnesses [22]. The Department of Health is responsible for the planning, funding, and quality of health policies in Catalonia. The Catalan Health Service (CatSalut) is responsible for commissioning the public health care system, which is organized in health regions. The 2006 Catalan Plan on Mental Health Care was being implemented when this study was conducted [23]. Girona was divided into 7 health areas, altogether, consisting of 221 municipalities. The Girona area had 0.7 million inhabitants (0.6 million adults), and its capital city had 96,000 inhabitants. The main public entity responsible for the provision of health and social care in Girona was the Institut D’Assitencia Sanitaria, which belonged to the Generalitat of Catalonia and acted through consultation with the Department of Health and the Department of Social Welfare and Family. Primary care was organized according to 36 basic territorial units. There was a general hospital in each health area that provided emergency and specialized care. In addition, there was a reference hospital that provided support to the hospitals in the health area and provided specialized and tertiary treatments for the Girona region. The MHS provided by the Institut D’Assistencia Sanitaria were divided into two principal areas: Hospital care, which was located in a health precinct named “Marti i Julia Hospital Park”; and community care, which was organized in the seven areas. Each of them was composed of an adult mental health center, a child-adolescent center, a drug addiction center, a rehabilitation community center, and residential resources (flats with specialized support) for mental health patients.

A group of socio-demographic indicators were selected to describe both study areas. Indicators were selected based on the European Socio-Demographic Schedule (ESDS) for the description of small mental health areas through demographic characteristics associated with mental disorders [24]. Besides this, other national indicators on incomes and health expenditures have been collected from the Organization for Economic Collaboration and Development (OECD). The selected indicators were: Gross domestic product (GDP) per capita in purchasing power standards (PPS) (where the EU28 average is set to equal 100% GDP for health expenditure), public and private health expenditure per capita in purchasing power parity (PPP), population mean age, percentage of female population, population density, unemployment rate, dependency ratio, percentage of single households, percentage of people on disability pension due to mental health disorders (percentage of all disability pensions), percentage of single-parent families, percentage of migrant population, overall mortality, and suicide mortality. The indicators were collected from Eurostat 2011 [25], World Health Organization [26], OECD Health Data 2011 [27], Sotkanet Statistics and Indicator Bank and Statistics Finland (for Helsinki and Uusimaa) [28], and the Statistical Institute of Catalonia-Idescat (for Girona) [29].

In both areas, MHS were mapped using the DESDE-LTC tool (Description and Evaluation of Services and Directories in Europe for Long Term) [30]. This mapping system was developed from the European Service Mapping Schedule (ESMS), which was developed to classify and standardize the mapping of adult mental health services in Europe [31]. The DESDE-LTC tool adapted and extended to long-term care (e.g., child and adolescents, drug and alcohol, disabilities, elderly, and chronic illness) the previous classification system provided by the ESMS for mental health care [32]. These instruments have been used in several previous studies that compared the mental health care provision: (i) In the same region or country [10,33,34], (ii) between divergent regions or districts in two countries [35,36,37,38], and (iii) in local areas across different countries in Europe [2].

The comparison of both MHS was based on the evaluation of the unit of analysis in the DESDE-LTC: The Basic Stable Input of Care (BSIC), and its’ code or label according to its Main Type of Care (MTC) [30]. BSICs are the minimal organizational units that have a stable team of personnel and provide care services on a routine basis to the same target population. They are identified by a set of standard operational criteria: Temporal stability (three years of functioning or sustainable budget) and organizational stability as defined by their placement capacity, target population, common professionals, and administrative autonomy (e.g., acute inpatient psychiatric unit in a general hospital). The MTC is a code provided by the DESDE-LTC taxonomy that describes the type of care provided by the BSIC. It allows grouping of similar units of delivery of care and differentiating them from other types of care (e.g., acute residential care and outpatient emergency care provided by the acute psychiatric inpatient unit in a general hospital).

The DESDE-LTC follows a hierarchical tree structure where, at level 0, the MTCs are divided into 6 main branches: Residential care (R), day care (D), outpatient care (O), information services (I), accessibility services (A), and self-help and voluntary care (S). The DESDE-LTC comprises over 90 different MTCs for the classification of the BSICs. For this comparative study, only the results on residential care, day care, and outpatient care were considered. Only services specialized in mental health were included. MHS for children were excluded from the mapping, i.e., only services primarily targeting people over 18 years of age were included. Forensic services and addiction services were likewise excluded.

The service mapping was performed by trained research assistants. Interrater reliability was ensured by a common training and tested by case vignettes. In both study areas, service mapping results were refined after consultation with local mental health care provision experts. In Girona, the mapping was carried out in 2010 and revised in 2011–2012; and in Helsinki and Uusimaa, it started in 2011–2012.

Productivity was expressed as hospital inpatient days and outpatient visits per full-time equivalent (FTE) mental health professional per year. It was counted only for the specialized psychiatric services, since the data for the use of primary care services in Helsinki and Uusimaa was incomplete, and in Girona there were no primary care mental health personnel. The number of psychiatric hospital inpatient days and psychiatric outpatient visits were collected from the central organization of the psychiatric services in Helsinki and Uusimaa, and the Catalonian Health Department for Girona. The number of occupied beds was calculated from the number of inpatient days per year.

## 3. Results

Table 1 describes the socio-economic characteristics and health expenditures of the study areas. The GDP and the health expenditures per capita (PPP) were higher in Finland, but the percentage of the GDP was greater in Spain. Both areas were over their respective GDP country average, which indicates that both are economically developed regions within their respective countries. Girona presented higher rates of unemployment and a higher immigrant population, whereas the higher income and educational level in Helsinki and Uusimaa was combined with higher rates in single households and single-parent households. Another indicator closely related to mental health status is suicide mortality, for which the rate in Helsinki and Uusimaa was 1.6 times as high as in Girona. The indicators related to gender and age characteristics were very similar in both areas.

The service mapping identified 246 BSICs and 265 MTCs in the Helsinki and Uusimaa region (Table 2). In Girona, there were 39 BSICs and 40 MTCs. In Helsinki and Uusimaa, there were 75.6 psychiatric hospital beds per 100,000 adults, and in Girona there were 22.4 beds. The number of non-hospital beds per 100,000 adults was 150.8 in Helsinki and Uusimaa and 21.7 in Girona. In day care services, there were 47.5 places per 100,000 adults in Helsinki and Uusimaa and 76.7 in Girona.

**Figure 1:**
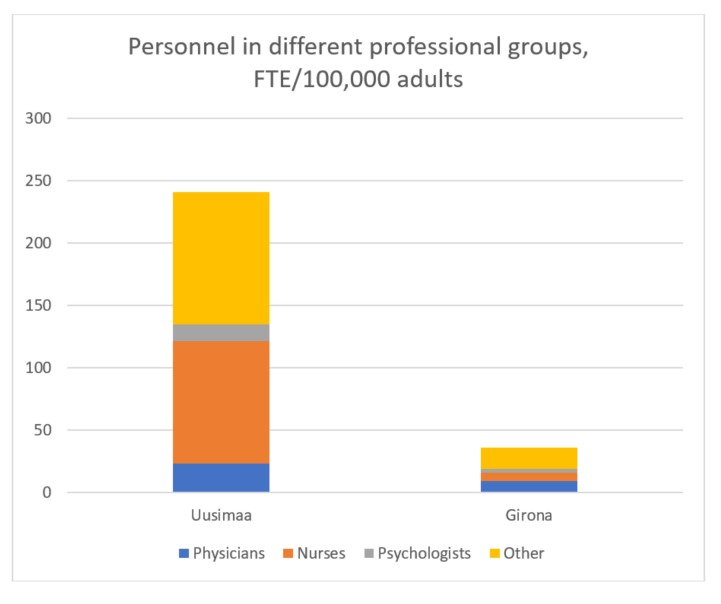
Personnel in different professional groups, expressed as full-time equivalents (FTE)/100,000 adults.

The total number of personnel in the MHS was 6.7 times higher in Helsinki and Uusimaa than in Girona (240.8 vs. 34.5 per 100,000 adults) (Table 3). There were 23.4 physicians in the MHS per 100,000 adult inhabitants in Helsinki and Uusimaa compared to 9.4 in Girona. The number of psychologists per 100,000 was 13 in Helsinki and Uusimaa and 2.9 in Girona. The biggest difference was found in the number of nurses: There were 98.3 nurses per 100,000 adults in Helsinki and Uusimaa compared to 6.5 per 100,000 in Girona (Figure 1).

**Figure 2:**
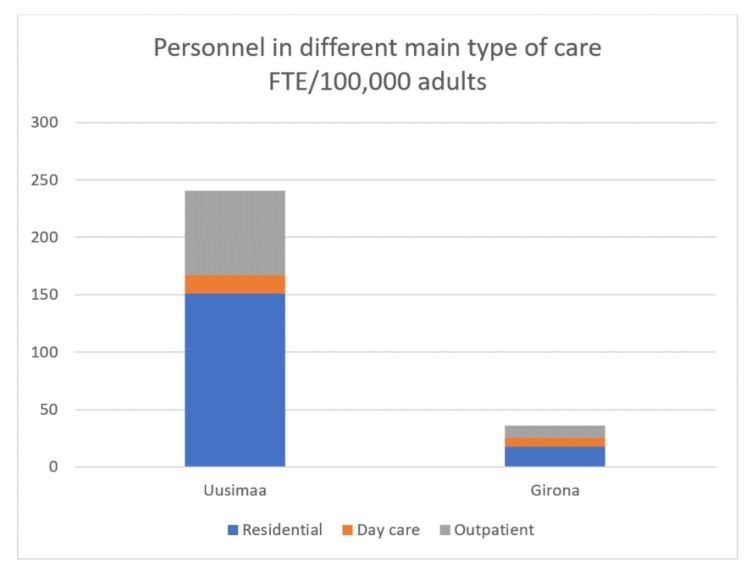
Personnel in different main types of care, expressed as full-time equivalents (FTE)/100,000 adults.

Regarding the total FTEs per main type of care, in Helsinki and Uusimaa, 62.7% of the total workforce capacity was allocated to residential care, while outpatient care received 30.7% and day care services received 6.6%. In Girona, 49.1% of the personnel was allocated to residential care, 29.2% to outpatient care, and 21.7% to day care (Figure 2).

The difference in the productivity of psychiatric hospitals and outpatient services per yearly FTE is presented in Table 4. The number of psychiatric hospital inpatient days per FTE per year was 231.3 in Uusimaa and 309.7 in Girona. Expressed as the number of FTE per occupied hospital bed, in Helsinki and Uusimaa, 1.58 FTE per bed were found, and, in Girona, 1.18. In specialized outpatient services, the number of visits per FTE was 506.8 in Helsinki and Uusimaa, and in Girona, it was 1110.6. Therefore, the outpatient personnel in Girona each handled 2.2 times as many patient visits as their colleagues in Helsinki and Uusimaa.

## 4. Discussion

In this standard comparison study, we found a remarkable difference in the population-adjusted mental health personnel resources in two Western European areas, with several similarities in their sociodemographics. There were 6.7 times more personnel in the MHS in the Helsinki and Uusimaa region compared to the Girona region. There were 2.5 times more physicians, 4.5 times more psychologists, and 15.1 times more psychiatric nurses in Helsinki and Uusimaa than in Girona. The productivity of outpatient visits per FTE in Girona was over twice the rate observed in Helsinki and Uusimaa. The suicide rate was 1.6 times higher in Helsinki and Uusimaa compared to Girona.

This study provides support from the mental health sector for the claim on the need for context analysis in complex interventions [41]. Understanding of the context and mental health care ecosystem is needed prior to implementing new service provision approaches in other environments. Previous findings [2,35,37,42] support the usability of making standard comparisons of MHS within regions, and across different countries, to guide management and policy planning. Even in the European context, significant differences exist in the structure and resourcing of MHS.

The prevalence of mental health disorders in Spain and Finland is approximately the same [43,44]. Mental health disorders are generally associated with a socially disadvantaged position, i.e., lower education, unemployment, social isolation, loneliness, and poorer material circumstances [45,46,47]. Scandinavian countries, like Finland, have lower poverty rates, smaller income differences, and higher self-rated health than southern European countries, such as Spain [48]. The effect of socioeconomic status has a stronger effect upon the self-rated health in Scandinavia than in southern Europe [49,50].

In our study, the Girona population showed lower average education, a higher unemployment rate and proportion of immigrants, a lower GDP, and a lower suicide rate than in Helsinki and Uusimaa. Suicidality has a complex etiology, including psychopathological, sociocultural, demographical, and geographical components. Social factors that are associated with an increased risk of suicide include living alone, financial or legal difficulties, and interpersonal stressors. The decrease in the risk of suicide is associated with a strong social network and religiosity [51]. However, the pattern of the mental health service organization also has significance regarding suicidality, with a more outpatient-care-oriented service structure being associated with a lower suicide rate [52]. The suicide rate has decreased in Finland by 41% between the years 1994 and 2015 [28]. At the same time, the number of psychiatric hospital inpatient days has decreased by 54% [28]. Still, the pattern of service provision in Finland is more hospital-based compared to other European countries [2].

The expenditure to health care, expressed as a percentage of GDP, is greater in Spain than in Finland. There are 380 physicians per 100,000 inhabitants in Spain and 300 in Finland [26]. However, according to our study, the investment in mental health care is low.

The biggest difference in the mental health service capacity was in residential services, both in the hospital and non-hospital contexts. Previous studies show that the use of psychiatric hospital inpatient care is associated with sociodemographic indicators for the area, such as the percentage of single households and use of alcohol [12,53,54,55,56]. The Helsinki and Uusimaa region showed more than double the rate of single households compared to Girona, which may increase the need for residential care services. In northern European countries, people leave their parental home earlier than in southern European countries [57,58]. In 2011, the estimated mean age of young people leaving the parental home in Finland was 22 years, while in Spain it was 29 years [59]. Cultural differences also influence the social acceptability of living alone vs. living with parents. While in Mediterranean countries, living at home until marriage is socially accepted, in northern European countries, young people leave home earlier without taking marriage into consideration [60]. In northern European countries, the role of the family is weaker and parents encourage young adults to leave home, while in strong-family areas, like Spain, the concept of the family as a group is predominant in the socialization process. Strong-family societies have higher social cohesion than weak ones and loneliness is an important social problem in weak-family areas [61].

In Spain, the family has a more prominent role in the support network for people in situations of dependency [15,62]. In Spain, 77.6% of schizophrenia patients live with their families; the primary caregiver in 63.4% of the cases being the parents, most often the mother [63]. Of these caregivers, 65.4% do not work outside of the home. This informal care adds to the financial burden of severe mental disorders [64]. According to a study in Finland, approximately 31% of the schizophrenia patients lived with their families––half of them with the parents [65]. The downsizing of psychiatric hospitals in Finland has led to trans-institutionalization to non-hospital residential services [2,14].

The population-adjusted capacity of the day care services was higher in Girona than in Helsinki and Uusimaa, but the personnel resources were higher in the Helsinki and Uusimaa region. The mental health care provision in Girona follows a community-based approach [2], which may be related to the Psychiatric Reform and the consequent community-based care delivery [16].

In general, the productivity of the services per FTE in Girona was higher than in Helsinki and Uusimaa. This may reflect a more efficient use of the sparse resources in Girona, but it may also mean a lower quality of care, with less time for direct contact with the patient. This subject merits further investigation, which should incorporate the analysis of key performance indicators and patient reported outcomes in both regions.

In both Finland and Spain, the mild and intermediately severe anxiety and affective disorders are treated in primary care. The mental health care received from primary care in Spain is mainly medication, with low resources for psychosocial support. Primary care in Spain has a gatekeeping role, controlling the access to specialized mental health care [66]. As an addition to the services that were mapped, in Finland the government funds psychotherapy provided by private psychotherapists. Part of the costs of psychotherapy remains to be covered by the patient. In Spain, no governmental support for private psychotherapy exists.

It is important to note that the Girona model has been developed in a location with a previous large psychiatric institution (Hospital Psiquiatrico de Salt). The analysis of other local areas with old psychiatric hospitals, such as Reus in Catalonia [34] or Gipuzkoa in the Basque Country [42], indicates that the local history of health care provision has a significant impact in the development of the mental health reform when compared with neighboring districts or regions. In Girona, the reform process did not involve the closure of the psychiatric hospital and a capital transfer of funding, but the incorporation of this facility into the main general health precinct of the region. A similar process has occurred in the psychiatric hospitals of Sant Boi in Barcelona [34].

In both the Helsinki and Uusimaa region and in Girona, the results of the REFINEMENT study have been considered in developing the local MHS. In Girona, the development of the community-oriented service system continues [2]; and in Helsinki and Uusimaa, three of the eight psychiatric hospitals have been closed and resources have partly been re-allocated. While in Finland, the challenge is to continue with true deinstitutionalization and to use effectively the available resources, in Spain, the challenge for the MHS seems to be the under-provision of resources. Currently, new studies on the mental health care provision using a similar method are being independently conducted in both areas to assess longitudinal changes in their MHS.

The comparison of service availability and the systems’ capacity (beds, places and workforce) provided in this study contributes to the organizational knowledge on the health care system, but it is not sufficient for local planning and actual resource allocation. As shown by the REFINEMENT study, planning requires additional information on financing, quality, and pathways of care. This information should also be completed by a standard description of the health care interventions or modalities of care provided in every service by the care teams (e.g., psychotherapy). The analysis of interventions requires other tools, such as the International Classification of Mental Health Care (ICMHC) [67] or the International Classification of Health Interventions (ICHI) [68]. In addition, resource allocation and local planning requires an analysis of the relative technical efficiency of the MHS. This is particularly relevant for future research as the study areas have shown a huge variability in their care provision systems. The efficiency analysis would eventually require data on clinical and patient reported outcomes. The collection of this information should be a priority for the departments of health in the two countries. It is also important to explore the patterns of horizontal integration across different sectors (health, social, employment, education, housing, and justice) and the vertical care integration of primary care, specialized community care, and hospital care. The departments of health have recognized the need to maximize integrated care in the two countries.

## 5. Conclusions

A major aspect of this research is to understand contextual factors and the health care ecosystem for analyzing the transferability of the results of any complex intervention to other local systems.

The results of the REFINEMENT study reveal that the MHS in two Western European societies show remarkable differences, part of which may reflect historical and sociocultural differences. The local variation of service provision identified in this study raises issues on how we should understand the variability of service provision in mental health, and the impact of this variation in the transferability of information on the effectiveness of interventions and service delivery models. It also raises issues on how to interpret key performance indicators in different environments or the actual value for local planning of the performance indicators provided by international organizations.

It is important for policy planning to have a standard comparison of the mental health care systems to continue with true deinstitutionalization, to develop more community-oriented services, and to ensure equal access to care in all European countries.

## Figures and Tables

**Table 1:** Socioeconomic characteristics of the studied areas.

Socioeconomic Indicator	Helsinki and Uusimaa	Girona
GDP per capita in PPS Index (EU-28 = 100) ^1^	155 (Helsinki and Uusimaa) 117 (Finland)	108 (Girona) 93 (Spain)
% GDP for health expenditure ^2^	9.0% (Finland)	9.5% (Spain)
Public and private health expenditure per capita ^2^	3486 US$ PPP per capita (Finland)	3019 US$ PPP per capita (Spain)
Mid-year population over 18 years of age ^3^	1,221,596	600,368
Population mean age (years) ^3^	39.5	40.4
Gender (% female) ^3^	51.6%	49.6%
Population Density (inhabitants per km^2^) ^3^	177	124
Unemployed (% of population 18–64 years) ^3^	7.0%	22.18%
Dependency ratio ^3^	46	47
Single households (% of all households) ^3^	41.4%	17.9%
People on disability pension due to mental disorders (% of all disability pensioners) ^3^	49.2%	30.9%
Single parent families (% of all families) ^3^	22.5%	7.7%
Migrant population (% of all residents) ^3^	8.9%	21.4%
Overall mortality (per 100,000 residents) ^3^	727	754
Suicide mortality per 100,000 adults ^4^	16.3	10.0
Population aged 25–64 with upper secondary or tertiary education completed ^5^	85.4%	54.9% (macro-area)

^1^ Finnish study area data and Spanish macro-area (Catalonia) data from Eurostat, 2011 [25]; ^2^ Whole country data, WHO estimates 2011 [26]; ^3^ Study area data from SOTKAnet Statistics and Indicator and STAT Finland 2011 (Helsinki and Uusimaa) [28] and Statistical Institute of Catalonia-Idescat, 2011 (Girona) [29]; ^4^ Average number of suicides in years 2011–2013 per 100,000 adults, Helsinki and Uusimaa region data from Statistics Finland, Girona data from Official statistics website of Catalonia [39]; ^5^ Finnish study area data and Spanish macro-area (Catalonia) data from Eurostat, 2011 [40].

**Table 2:** The main type of care (MTC) units and capacity per 100,000 adults in residential care, day care, and outpatient service branches of the Description and Evaluation of Services and Directories in Europe for Long Term (DESDE-LTC) code tree.

Main Type of Care	DESDE- LTC Code	MTC Units/100,000 Adults (Absolute Numbers in Brackets)		Beds/Places/Users /100,000 Adults (Absolute Numbers in Brackets)	
		Helsinki and Uusimaa	Girona	Helsinki and Uusimaa	Girona
Residential care					
Hospital, acute care	R1–3	1.64 (20)	0.17 (1)	23.74 (290 beds)	7.01 (42 beds)
Hospital, non-acute	R4, R6	3.27 (40)	0.33 (2)	51.82 (633 beds)	15.35 (92 beds)
Non-hospital	R5, R7	0.33 (4)	0	3.77 (46 beds)	0
Non-physician cover, 24 h support	R8, R11	3.36 (41)	0.17 (1)	100.28 (1225 ** beds)	9.68 (58 beds)
Non-physician cover, non-24 h support	R9–10, R12–13, R14	2.29 (28)	1.67 (10)	46.74 (571 beds)	12.01 (72 beds)
Total residential care	R	10.89 (133)	2.34 (14)	226.34 (2765 beds)	44.04 (264 beds)
Day care					
Acute day care	D1	0.65 (8)	0.17 (1)	9.50 (116 places)	4.2 (25 places)
Work-related day care	D2–3, D6–7	1.56 (19)	1.33 (8)	13.02 (159 *)	32.5 (195 ** places)
Health-related day care	D4.1, D8.1	1.15 (14)	0.50 (3)	19.40 (237 *)	12.5 (75 places)
Structured and non-structured day care	D4.2–D4.4, D5	0.33 (4)	0.83 (5)	5.57 (68)	27.5 (165 places)
Total day care	D	3.44 (42)	2.84 (17)	47.48 (580 * places)	76.73 (460 ** places)
Outpatient care					
Acute mobile care	O1–2	0.08 (1)	0	11.73 (143 users a month)	0
Acute care	O3–4	1.06 (13)	0.17 (1)	75.67 (924 users a month)	57.38 (344 users a month)
Non-acute, mobile care	O5–7	1.15 (14)	0.17 (1)	19.87 (243 users a month)	2.50 (15 users a month)
Non-acute	O8–10	5.08 (62)	1.17 (7)	220.51 (2694 ** users a month)	244.55 (1466 *** users a month)
Total outpatient care	O	7.37 (90)	1.50 (9)	327.78 (4004 ** users a month)	304.43 (1825 users a month)
MTCs total		21.69 (265)	6.67 (40)	273.82 (3345 * beds + places)	120.77 (724 ** beds + places)

* Some MTCs do not have a limited number of places. ** Places/users are not available for some MTCs.

**Table 3:** Personnel in full-time-equivalents (FTE) per profession and per main type of care.

	Helsinki and Uusimaa		Girona	
	FTE/100,000 Adults	FTE	FTE/100,000 Adults	FTE
Profession				
Physicians	23.4	286	9.4	56
Nurses	98.3	1201	6.5	39
Psychologists	13.0	159	2.9	17
Other (social workers, occupational therapists, auxiliary nurses)	106.0	1295	17.2	103
Total	240.8	2941	36.0	216
Total FTEs per main type of care				
Residential	151.0	1845	17.7	106
Day care	15.9	194	7.8	47
Outpatient	73.8	902	10.5	63
Total	240.8	2941	36.0	216

**Table 4:** The productivity of specialized psychiatric care expressed as the number of outpatient visits and hospital inpatient days per FTE and the number of FTE per occupied psychiatric hospital bed.

Productivity	Helsinki and Uusimaa	Girona
Outpatient visits in specialized psychiatric care per FTE (Absolute number in brackets)	506.8 (409,573)	1110.6 (69,967)
Psychiatric hospital inpatient days per FTE (Absolute number in brackets)	231.3 (277,675)	309.7 (28,490)
Number of FTE per occupied psychiatric hospital bed (Absolute number of occupied beds in brackets)	1.58 (760.8)	1.18 (78.1)
